# Perceptions of inequality and meritocracy: their interplay in shaping preferences for market justice in Chile (2016–2023)

**DOI:** 10.3389/fsoc.2025.1634219

**Published:** 2025-09-18

**Authors:** Juan Carlos Castillo, Andreas Laffert, Kevin Carrasco, Julio Iturra-Sanhueza

**Affiliations:** ^1^Departamento de Sociología, Universidad de Chile, Santiago, Chile; ^2^COES - Center for Social Conflict and Cohesion Studies, Santiago, Chile; ^3^Departamento de Sociología, Pontificia Universidad Catolica de Chile, Santiago, Chile; ^4^Bremen International Graduate School of Social Sciences, Bremen, Germany

**Keywords:** inequality perception, meritocracy, commodification, Chile, public preferences

## Abstract

**Introduction:**

Several countries have experienced a shift toward the privatization and commodification of public goods, welfare policies, and social services. In Latin America, Chile stands out as a paradigmatic case where this trend has led to the extensive marketization of essential services. From a moral economy perspective, the extent to which individuals consider it fair for access to such services to depend on market criteria has been conceptualized as market justice preferences. This study investigates the relationship between perceptions of economic inequality, meritocratic beliefs, and market justice preferences in Chile between 2016 and 2023.

**Methods:**

Using six waves of panel data from the Chilean Longitudinal Social Survey-ELSOC (Nobservations = 8,643; Nindividuals = 1,687), the analysis examines how subjective assessments of inequality shape attitudes toward the role of merit in access to key social services such as healthcare, education, and pensions.

**Results:**

The findings show that greater perceived inequality is associated with lower market justice preferences. However, individuals who believe that effort is rewarded are more likely to legitimize existing disparities. In contrast, the perception that talent is rewarded shows a negative effect on market justice preferences; an effect that intensifies as perceived inequality increases over time. The study also considers the influence of major social movements during this period, which appear to have reshaped public discourse on justice and fairness.

**Discussion:**

These findings contribute to a deeper understanding of how distributive beliefs evolve in contexts marked by persistent inequality and entrenched neoliberal frameworks. They indicate that while perceptions of inequality tend to undermine support for market justice, meritocratic beliefs-particularly those emphasizing effort-reinforce its legitimacy. By contrast, talent-based meritocratic perceptions weaken it, especially as inequality becomes more salient. The results also suggest that major collective events, such as the 2019 protests, did not fundamentally alter these underlying associations.

## 1 Introduction

Since 1980, economic inequality and wealth concentration have dramatically increased worldwide, becoming one of the main challenges for the social sciences. Globally, in 2021, less than 50% of the world population owned only 2% of the wealth, while the richest 10% concentrated 76%. Futhermore, the wealthiest 1% captured nearly 38% of total assets (Chancel et al., 2022). This context of economic disparity has sparked renewed interest in studying not only the objective aspects of inequality, such as income and access to resources, but also its subjective dimensions, including perceptions, beliefs, and associated attitudes (Janmaat, 2013). Such dimensions are deemed as key to understand the delegitimation of political institutions that economic inequality can elicit (Castillo et al., 2022). Therefore, understanding how people perceive inequality is crucial, as these perceptions can influence how societies comprehend and justify (or not) inequality in the distribution of goods and services, as well as their capacity to funnel political discontent and eventually opposition to the current economic distribution.

The justification of economic inequality is a topic with a long tradition in sociology, with seminal works such as the ones from Kluegel et al. (1995), Kluegel and Smith (1986) and Lane (1962, 1986). Lane's (1986) main aim was to “help explain the tenacious hold of market capitalism on the public mind”, proposing the distinction between market and political justice. Whereas political justice refer to government actions aligned with justice principles such equality or need, market justice involves the allocation linked to earned deserts, particularly through individual contributions based on individual merit. Part of this agenda has focused on analyzing the extent to which individuals consider as just to get better access to social services (such as pensions, health and education) based on payment capacity (Busemeyer, 2014; Castillo et al., 2024; Immergut and Schneider, 2020; Koos and Sachweh, 2019; Lindh, 2015; Lindh and McCall, 2023), usually under the label of market justice preferences. Although these services can be considered fundamental rights of citizenship, existing evidence indicates that a substantial share of the population legitimizes inequality in these domains, and that such preferences are shaped by individuals' socioeconomic characteristics as well as the broader contextual features of their countries (Busemeyer, 2014; Koos and Sachweh, 2019; Lindh, 2015). Within this framework, we argue that there are two relevant factors that are usually sidelined in this literature and that are key to understand market justice preferences: the perception of economic inequality and the perception of meritocracy.

The perception of economic inequality can be understood as an individual's subjective assessment of how resources are allocated among members of a given society (Akyelken, 2020). Regardless of their measurement, various studies have shown that this perception often underestimates the gap between the rich and the poor, revealing inconsistencies between objective and subjective aspects of inequality (Trump, 2018). Moreover, there is consistent evidence that perceived inequality, even under control of objective inequality, could have implications for attitudes toward the distribution of goods and services (Castillo et al., 2022; Schröder, 2017), redistributive preferences, justification of inequality, and legitimacy of the economic system (Castillo et al., 2022; García-Sánchez et al., 2020, 2018). From this perspective, in this paper we argue that perceptions of economic inequality would play a relevant role for understanding market justice preferences, as a lower perceived inequality could reinforce the idea that the differences in access to social services could be justified—a link that becomes harder to hold when high inequality is perceived.

Along with perception of inequality, a second focus of this paper is the perception of meritocracy. Meritocracy posits that inequality can be legitimized through distributive criteria such as effort and talent (Davis and Moore, 2001; Young, 1962). Previous studies have shown that those with stronger meritocratic perceptions tend to justify larger inequality, as economic differences are legitimized by being attributed to individual achievements (Batruch et al., 2023; Mijs, 2021; Wilson, 2003). We argue that in contexts where the distribution of goods and services is predominantly governed by market logics, as in the chilean case (Boccardo, 2020; Madariaga, 2020), meritocratic perceptions could play a role in the preferences for market justice.

The primary objective of this study is to analyze the interplay between perceptions of inequality and meritocracy and their joint influence on preferences for market justice along time in Chile using the panel survey ELSOC (Social Chilean Longitudinal Study, 2016–2023). Furthermore, we explore how political and social contingencies—such as the 2019 and 2022 social movements—might have moderated these relationships by prompting more critical reflection on the commodification of social services (Somma et al., 2021). Recognizing the temporal dimension in shaping preferences is essential, given that historical and contextual factors that could challenge or reaffirm the way in which individuals perceive inequality and meritocracy, resulting in changes in market justice preferences.

## 2 Market justice preferences

Conceptually, *market justice* has been discussed in the literature as a normative principle that legitimates the distribution of economic rewards based on individual merit. It is possible to trace the concept to the understanding of Lane (1986), who makes a contrast between market justice and political justice. The author defines market justice as a system of “earned deserts”, whereby individuals are seen as deserving of a determined distributive outcome due to their effort and skills. In contrast, political justice emphasizes principles of equality and need, which are often represented by the welfare state action through social policies. An important remark is that the principles of market justice rely on the assumption that markets are neutral and self-regulating arenas, where individuals are treated fairly because they face the same formal rules of engagement and procedures (Lane, 1986). Consequently, the legitimacy of market justice stems from the assumption that inequalities are not only inevitable but fair—so long as the rules are transparent and opportunities are open. In this way, market justice provides a moral justification for inequality by framing it as a necessary outcome of individual responsibility (Kluegel et al., 1999; Lindh, 2015).

Empirical studies have shown different strategies for the study of market justice preferences. A common approach in the literature is to gauge attitudes toward the legitimacy of inequality in specific domains, especially when linked to income differences. This can be traced to the seminal work of Kluegel and Smith (1981) who assessed the normative foundations that explain public support for economic inequality. Over time, this approach has been extended beyond income to include other market-mediated outcomes, such as education, healthcare, and/or pensions. For example, Von Dem Knesebeck et al. (2016) and Immergut and Schneider (2020) examine whether citizens consider it fair that individuals with higher incomes can access better healthcare, while Lee and Stacey (2023) apply a similar method in the context of education in Australia. These studies usually rely on survey items asking respondents to evaluate the fairness of income-based access to welfare and social services (Lindh, 2015; Svallfors, 2007). More recently, Castillo et al. (2024) introduced a single-item composite measure of market justice to assess student attitudes toward income-based access to education, healthcare, and pensions in Chile. Such empirical strategies aim to capture the extent to which individuals accept inequality when framed as a reflection of market outcomes.

The study of market justice preferences has increasingly focused on how they are shaped by individuals' socioeconomic position, normative beliefs, and the institutional context in which they are embedded (Pierson, 1993). Across the literature, there is empirical evidence suggesting that individuals in more advantaged socioeconomic positions—those with higher occupational class, income, and education—are more likely to support market justice principles (Koos and Sachweh, 2019; Lindh, 2015; Svallfors, 2007). This tendency reflects not only material self-interest but also a broader moral economy, in which winners of the market system internalize justifications for the status quo. At the same time, political ideology also plays a role—such as economically conservative values—where right-wing individuals show higher support for meritocracy and more skepticism toward redistribution. This is particularly salient in countries with more restricted public provision of social services. For example, in cases such as Chile (Castillo et al., 2024) and Australia (Lee and Stacey, 2023) the evidence suggests that right-wing individuals are more supportive of market-based distribution of welfare. Regarding contextual factors, in liberal welfare regimes like those of the United States or the United Kingdom, market justice preferences are more widespread, while in coordinated or social-democratic regimes—such as Sweden or Germany—citizens are generally more critical of market-based inequalities (Immergut and Schneider, 2020; Lindh, 2015).

## 3 Perceptions of inequality and meritocracy

### 3.1 The perception of economic inequality

Perceptions of inequality refer to individuals' subjective evaluations of the extent, causes, and consequences of income and wealth disparities (Akyelken, 2020). Unlike objective measures such as the Gini index, perceived inequality captures how individuals make sense of distributive hierarchies in their everyday lives, shaped by reference groups, social comparisons, and information environments (García-Castro et al., 2020; Gimpelson and Treisman, 2018; Mijs, 2016a). Scholars have proposed multiple dimensions of perceived inequality, including its magnitude (how significant are the gaps), vertical structure (between which groups), the trend over time (increasing or decreasing), and legitimacy (whether it is just or not) (Engelhardt and Wagener, 2018; García-Sánchez et al., 2019). These dimensions encompass both cognitive and normative aspects of perceptions of inequality and can vary across societies and social groups, depending on exposure, ideology, and personal experience (Castillo et al., 2022; García-Sánchez et al., 2018).

Perceptions of inequality have been associated with a range of distributive attitudes (Castillo et al., 2022; García-Sánchez et al., 2019; McCall et al., 2017; Trump, 2018). Research indicates that perceptions of inequality influence redistributive preferences to a greater extent than objective or actual economic inequality (Castillo, 2011; Gimpelson and Treisman, 2018). Lower perceived inequality can reinforce support for market-based distributive arrangements (Kuhn, 2011). In contrast, when inequality is perceived as excessive, individuals could question the legitimacy of market justice and become more supportive of redistributive policies (García-Sánchez et al., 2019).

Assessing the perception of inequality in empirical research is an ongoing challenge. Specifically in survey research, the assessment of perceived inequality through Likert-type items such as “income differences are too large” have been questioned as they captures general concern or discomfort rather than a specific perception (Castillo, 2011; García-Sánchez et al., 2019). As a result, the conceptual clarity between perceived inequality and inequality aversion remains blurred in many empirical studies. To address this limitation, recent work has emphasized the need to distinguish between absolute and comparative measures, as well as between ideal and perceived estimates of economic gaps (Auspurg et al., 2017; García-Sánchez and De Carvalho, 2022). Through asking perceived salaries for occupations in the extremes of the status continuum (such as a CEO and a manual worker), an indirect measure of perceived inequality is built by the ratio of the high and the low status occupation. This type of measure provides a concrete frame that respondents can relate to more easily than abstract questions about national income distribution (Castillo et al., 2012; Easterbrook, 2021; Willis et al., 2015). Evidence to date shows that perceived wage gaps are strong predictors of political attitudes (García-Sánchez et al., 2018; Pedersen and Mutz, 2019), making them a valuable tool for understanding public responses to economic disparities.

### 3.2 Perception of meritocracy

Meritocracy constitutes a central ideological framework for legitimizing various forms of social inequality (Mijs, 2016b; Wilson, 2003). Rooted in the belief that rewards and social positions should be allocated based on individual effort and talent, meritocracy operates both as a descriptive account of how societies function and as a normative ideal of how they ought to function. As originally conceptualized by Young (1962), the term was intended as a critique of a system in which merit-based stratification gives rise to a new form of inequality. However, over time, meritocracy has come to be widely endorsed as a fair and desirable principle of distribution, particularly within liberal democracies and market-oriented societies (Mijs, 2021; Sandel, 2020).

From a sociological perspective, belief in meritocracy extends beyond a cognitive judgment; it constitutes a moral lens through which individuals interpret social and economic disparities (Mijs, 2021). Individuals who believe that success results from hard work and talent are more likely to regard inequality as legitimate (Batruch et al., 2023; Castillo et al., 2012; Tejero-Peregrina et al., 2025). In contrast, when outcomes are attributed to luck, social origin, or structural barriers, inequality is more often perceived as unjust (Hauser and Norton, 2017; Reynolds and Xian, 2014). This distinction becomes especially salient in societies marked by persistent structural inequality, where dominant narratives emphasize personal responsibility while neglecting the role of entrenched disadvantage.

Recent research has highlighted the importance of disentangling the concept of “meritocratic beliefs,” particularly by distinguishing between meritocratic preferences and meritocratic perceptions (Castillo et al., 2023). While preferences refer to normative ideals about how rewards should be allocated (i.e., based on merit), perceptions refer to subjective evaluations of how meritocracy actually operates in practice (i.e., how rewards are allocated) (Janmaat, 2013). This distinction allows for a more precise understanding of how individuals make sense of inequality: people may endorse meritocracy normatively while simultaneously recognizing its failure in practice. In stratified societies, the gap between preferences and perceptions is particularly evident, as individuals may continue to uphold the ideal of meritocracy despite growing awareness of unequal opportunities (Mijs et al., 2022).

Within the domain of meritocratic perceptions (Castillo et al., 2023), we further distinguish between two core dimensions: effort-based and talent-based perceptions. This distinction is analytically important, as it captures different mechanisms through which individuals may justify inequality (Reynolds and Xian, 2014; Young, 1962). Effort-based perceptions emphasize hard work and perseverance as the key drivers of success, aligning closely with cultural narratives of individual responsibility and moral deservingness (Dubet, 2011). In contrast, talent-based perceptions emphasize innate ability and intelligence—traits often regarded as less malleable and more unequally distributed (Young, 1962). We argue that this latter dimension may shape attitudes differently, as talent tends to be seen as a given attribute rather than the result of personal effort.

Meritocratic perceptions function as a normative framework that legitimizes unequal outcomes, particularly when access to social goods is stratified by income or socioeconomic background. Prior studies have consistently shown that individuals who perceive society as meritocratic express lower support for redistribution (Hoyt et al., 2023; Tejero-Peregrina et al., 2025), greater legitimization of class inequality (Batruch et al., 2023), increased tolerance for inequality (Day and Fiske, 2017), and lower perceptions of income inequality itself (Castillo et al., 2012). Regarding market justice preferences, prior studies in Chile have shown that individuals who perceive higher levels of meritocracy tend to express stronger support for unequal distributions that reflect market outcomes in healthcare, education and pensions (Castillo et al., 2024). Taken together, and as Busemeyer et al. (2021) argues, meritocratic narratives can operate as feedback mechanisms that shape public opinion and individual well-being by framing welfare outcomes as either deserved or undeserved. Such mechanism underscores the normative power of meritocracy in stabilizing unequal systems by shaping political attitudes and personal understandings of success and failure.

### 3.3 The Chilean context

Chile offers an interesting case for examining how public attitudes toward the distribution of social services evolve amid declining poverty and persistently high income inequality within a residual welfare framework (Ferre, 2023). Following the neoliberal reforms of the 1980s introduced during the civil-military dictatorship (1973–1989), the Chilean welfare state has become increasingly reliant on private provision, with essential services often commodified and largely reserved for those with sufficient purchasing power (Boccardo, 2020; Madariaga, 2020). This model has favored the affluent, while lower-income groups are left dependent on limited and underfunded public alternatives. Research on social stratification characterizes Chile as a society where mobility into intermediate classes is feasible, but access to elite strata remains largely restricted (López-Roldán and Fachelli, 2021; Torche, 2014). While some indicators—such as education and occupation—suggest a relatively fluid class structure, income mobility remains constrained (Espinoza and Núñez, 2014).

Despite steady economic expansion, Chile continues to rank among the most unequal nations in the OECD, marked by a high Gini coefficient and substantial wealth concentration at the top (Flores et al., 2020). In this context, Chile experienced a wave of mass protests from October 2019 to March 2020. Initially led by high school students, the demonstrations quickly gained momentum as broader segments of society mobilized around demands for more equality of opportunities, particularly regarding precarious access to welfare services. The political system interpreted this unrest as a call to reform existing political institutions, with a special focus on equalizing opportunities and providing more egalitarian access to welfare by reducing reliance on out-of-pocket mechanisms (Somma et al., 2021).

### 3.4 This study

Building upon the previous literature, this study proposes that attitudes toward market justice are shaped by a dynamic interplay between individuals' perceptions of economic inequality and their perceptions of meritocracy. Specifically, we argue that both perceptions independently and interactively influence the extent to which individuals endorse market-based distributions of social goods and services in Chile. To our knowledge, there are no studies so far analyzing such associations, let alone from a longitudinal perspective.

Firstly, and consistent with previous findings about research on redistributive preferences (Castillo et al., 2022), we expect that a higher perception of economic inequality will be associated with lower market justice preferences. When individuals perceive smaller income gaps, they are more likely to view market mechanisms as fair and legitimate, reinforcing the acceptance of outcomes based on competition and ability to pay. Conversely, a heightened perception of inequality may erode confidence in market fairness, weakening support for market-based distribution. This relationship is particularly relevant in the context of Chile, where the neoliberal economic model has been a dominant force in shaping public attitudes toward inequality and justice (Canales Cerón et al., 2021; Mac-Clure et al., 2024).

Second, higher perceived meritocracy is expected to be positively associated with market justice preferences. Individuals who believe that effort and talent primarily determine success would be more likely to justify unequal outcomes and endorse the notion that markets allocate resources fairly according to individual merit (Castillo et al., 2024). This aligns with the idea that meritocratic perceptions serve as a moral framework that legitimizes market-based inequalities, as individuals perceive the system as just when they believe that rewards are based on individual merit. This is particularly relevant in contexts where neoliberal ideologies dominate, as they often emphasize individual responsibility and competition as the basis for social order.

Third, we propose that perceptions of meritocracy and perceptions of economic inequality interact in shaping market justice attitudes. Specifically, we argue that the legitimizing effect of perceived meritocracy on market justice preferences is moderated by perceived economic inequality: when perceived inequality is low, the positive association between meritocratic perceptions and market justice preferences will be stronger. However, when perceived inequality is high, this association will weaken, as greater awareness of large economic gaps may challenge the view that outcomes are purely merit-based.

Additionally, this study examines whether these relationships vary over time, particularly in light of broader social and political transformations. We are especially interested in whether changes in individuals' perceptions of inequality and meritocracy are associated with shifts in their market justice attitudes across different time points. While we acknowledge the relevance of contextual events for public opinion and political orientation (Disi Pavlic et al., 2025), such as the 2019 political outburst or the subsequent constitutional process in Chile, we are aware that identifying causal associations lies outside the scope of this research. Instead, and from an exploratory perspective, we focus on how intra-individual trajectories in perceived inequality and merit shape evolving attitudes toward the commodification of social services.

Based on these arguments, we propose the following hypotheses:

*H*1_*a*_(between-person): individuals who perceive higher levels of economic inequality will report lower support for market justice preferences, on average, compared to those who perceive lower levels of inequality.

*H*1_*b*_(within-person): increases in an individual's perception of economic inequality over time will be associated with decreases in their support for market justice preferences.

*H*2_*a*_(between-person): individuals who endorse stronger meritocratic perceptions will report higher support for market justice preferences, on average, compared to those with weaker meritocratic perceptions

*H*2_*b*_(within-person): increases in an individual's meritocratic perceptions over time will be associated with increases in their support for market justice preferences.

*H*3_*a*_(between-person): the positive association between meritocratic perceptions and market justice preferences will be weaker among individuals who perceive higher levels of economic inequality, compared to those who perceive lower inequality.

*H*3_*b*_(within-person): the positive association between meritocratic perceptions and market justice preferences will be weaker at times when individuals perceive higher levels of inequality, relative to times when they perceive lower inequality.

*H*4_*a*_(growth moderation - inequality): the longitudinal association between perceived economic inequality and market justice preferences will strengthen over time, both within and between individuals.

*H*4_*b*_(growth moderation - meritocracy): the longitudinal association between meritocratic perceptions and market justice preferences will weaken over time, both within and between individuals.

## 4 Data, variables and methods

### 4.1 Data

This study draws on data from the Chilean Longitudinal Social Survey (ELSOC), a nationally representative panel study of the urban adult population in Chile, conducted annually between 2016 and 2023. Designed to examine individuals' attitudes, emotions, and behaviors regarding social conflict and cohesion, ELSOC employs a probabilistic, stratified, clustered, and multistage sampling design covering both major urban centers and smaller cities. The sampling frame was proportionally stratified into six categories of urban population size (e.g., large and small cities), followed by the random selection of households within 1,067 city blocks. The target population includes men and women aged 18 to 75 who are habitual residents of private dwellings.

The survey has been conducted every year since 2016, except in 2020, when it was suspended due to the COVID-19 pandemic. This study uses six waves: 2016, 2017, 2018, 2019, 2022, and 2023. The 2021 wave was excluded because a reduced version of the questionnaire omitted key variables of interest. Between waves 1 and 6, panel attrition reached 40%, resulting in a final two-level sample comprising N = 8,643 observations nested within N = 1,687 individuals. Longitudinal weights are applied to adjust for both the sampling design and potential biases from systematic non-response. Further details on sampling, attrition, and weighting procedures are available at https://coes.cl/encuesta-panel/, and the dataset is publicly accessible at https://dataverse.harvard.edu/dataverse/elsoc.

### 4.2 Variables

#### 4.2.1 Market justice preferences

The dependent variable in this study is preferences for market justice. This construct is operationalized through three items that capture how strongly individuals justify conditioning access to core services—healthcare, pensions, and education— basen on individual income. Specifically, the justification of inequality in healthcare is assessed by the question: “Is it fair in Chile that people with higher incomes can access better healthcare than people with lower incomes?” The same question is posed for pensions and education. In all cases, respondents indicate their level of agreement on a five-point Likert scale ranging from 1 (“strongly disagree”) to 5 (“strongly agree”). Additionally, we include a composite measure of “market justice preferences,” calculated as the average of these three items (α = 0.84). This index ranges from 1 to 5, with higher values indicating stronger preferences for market justice (see Table 1).

**Table 1:** Dependent variables for the first wave (2016).

**Label**	**Stats/values**	**Freqs (% of valid)**	**Valid**
Health distributive justice	1. Strongly disagree	558 (37.2%)	1,501(100.0%)
	2. Disagree	729 (48.6%)	
	3. Neither agree nor disagree	63 ( 4.2%)	
	4. Agree	133 ( 8.9%))	
	5. Strongly agree	18 ( 1.2%)	
Pension distributive justice	1. Strongly disagree	426 (28.4%)	1,501(100.0%)
	2. Disagree	718 (47.8%)	
	3. Neither agree nor disagree	108 ( 7.2%)	
	4. Agree	226 (15.1%)	
	5. Strongly agree	23 ( 1.5%)	
Education distributive justice	1. Strongly disagree	521 (34.7%)	1,501 (100.0%)
	2. Disagree	783 (52.2%)	
	3. Neither agree nor disagree	73 ( 4.9%)	
	4. Agree	113 ( 7.5%)	
	5. Strongly agree	11 ( 0.7%)	
Market justice preferences	Mean (SD) : 2 (0.8)	12 distinct values	1,501(100.0%)
	min < med < max:		
	1 < 2 < 5	
	IQR (CV) : 0.7 (0.4)	

#### 4.2.2 Perception of economic inequality

This variable is measured through the perceived wage gap (Castillo, 2009; Gijsberts, 1999; Hadler, 2005). This measure is derived from the salary gap between the perceived salaries of jobs at opposite ends of the occupational hierarchy. Specifically, it relies on the division between the perceived salary of a large-company president and that of an unskilled worker (Castillo, 2011). Higher values of this term indicate a greater perception of economic inequality between occupations located at the extremes of the status continuum. This measure includes a logarithmic term in order to adjust income magnitudes (usually fewer cases with high income):


$$\begin{array}{c} \text{perceived wage gap}=\\ \log_{10}\left(\frac{\text{perceived salary of a large-company president}}{\text{perceived salary of an unskilled worker}}\right) \end{array}$$

#### 4.2.3 Perception of meritocracy

This variable is operationalized through two components, namely effort and talent (Young, 1962). The item used to gauge effort is: “In Chile, people are rewarded for their efforts,” while the item for talent is: “In Chile, people are rewarded for their intelligence and skills.” In both cases, respondents indicate their level of agreement on a five-point Likert scale, ranging from 1 (“strongly disagree”) to 5 (“strongly agree”).

Table 2 shows the independent variables used, their response categories and their frequencies.

**Table 2:** Independent variables ELSOC survey (descriptives for first wave 2016).

**Label**	**Stats/values**	**Freqs (% of valid)**	**Valid**
Inequality gap perception	Mean (SD) : 3.7 (1.1)	296 distinct values	1,501 (100.0%)
	min < med < max:		
	0.4 < 3.7 < 6.9		
	IQR (CV) : 1.6 (0.3)		
People are rewarded for their efforts	1. Strongly disagree	169 (11.3%)	1,501 (100.0%)
	2. Disagree	693 (46.2%)	
	3. Neither agree nor disagree	263 (17.5%)	
	4. Agree	328 (21.9%)	
	5. Strongly agree	48 (3.2%)	
People are rewarded for their intelligence	1. Strongly disagree	134 ( 8.9%)	1,501(100.0%)
	2. Disagree	617 (41.1%)	
	3. Neither agree nor disagree	294 (19.6%)	
	4. Agree	401 (26.7%)	
	5. Strongly agree	55 ( 3.7%)	

#### 4.2.4 Controls

Sociodemographic and attitudinal variables are included to control for potential composition effects in the population. In terms of sociodemographic characteristics, we incorporate per capita household income quintile, educational level (1 = Less than Universitary, 2 = Universitary), age (in ranges), and sex (1 = Male, 2 = Female), which have been previously shown to influence market justice preferences significantly (Castillo et al., 2024; Lindh, 2015). Regarding attitudinal variables, we include political identification (1 = Left, 2 = Center, 3 = Right, 4 = No identification) and subjective social status (measured through a scale from 1 to 10) as they may affect the relationship between market justice preferences, perceptions of inequality, and meritocracy (Schneider and Castillo, 2015). Descriptive statistics for the control variables can be found in the Supplementary material.

### 4.3 Methods

Given the data's hierarchical structure, in which observations are nested in survey waves, we employ longitudinal multilevel linear models (Singer and Willett, 2009). In a panel-data framework, within-person effects capture how shifts in individual-level variables across waves are associated with variations in market justice preferences. By contrast, between-person effects focus on differences among individuals, explaining how long-term (or average) values relate to overall levels of market justice preferences.

To estimate within-person effects, we use group-mean centering, where each respondent functions as the “group” (i.e., observations nested within persons). Meanwhile, the between-person effects are derived from each individual's average on these variables, calculated across the waves of panel data.

All the analyses were conducted using R software and the *lme4* package (Bates et al., 2015).

## 5 Results

### 5.1 Descriptive statistics

**Figure 1:**
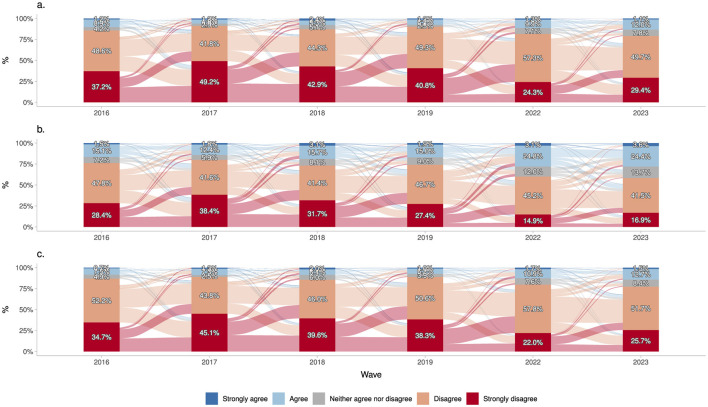
Change in the justification of inequality in healthcare, pensions and education over time (2016–2023). **(a)** Healthcare. **(b)** Pensions. **(c)** Education. Source: own elaboration with pooled data from ELSOC 2016–2023 (N obs = 8643; N groups = 1687).

Figure 1 shows the annual frequencies of market justice preferences for healthcare, pensions, and education from 2016 to 2023. Each year presents stacked percentage frequencies, and the flows between them reflect opinion changes among the same individuals from one year to the next, given that we are using panel data. For instance, of the 40.8% who strongly disagreed with justifying inequality in healthcare in 2019, around 24.3% maintained that position in 2022, while the remaining 16.5% shifted toward other response categories—primarily moving into disagreement rather than strong disagreement. Overall, more than half of the respondents exhibit a high level of disagreement (disagree + strongly disagree) with inequality in these three social service areas over time. Despite this general pattern, recent waves show a slight decrease in disagreement and a corresponding rise in support for market-justice inequality. Specifically, in healthcare and education, although disagreement remains substantial, agreement (agree + strongly agree) increased from 7.4 and 7.2% in 2019 to 13.1% and 14.2% in 2023, respectively. This shift is most evident in pensions, where the combined agree/strongly agree category grew by about 10 percentage points, from 16.9% in 2016 to 28% in 2023.

**Figure 2:**
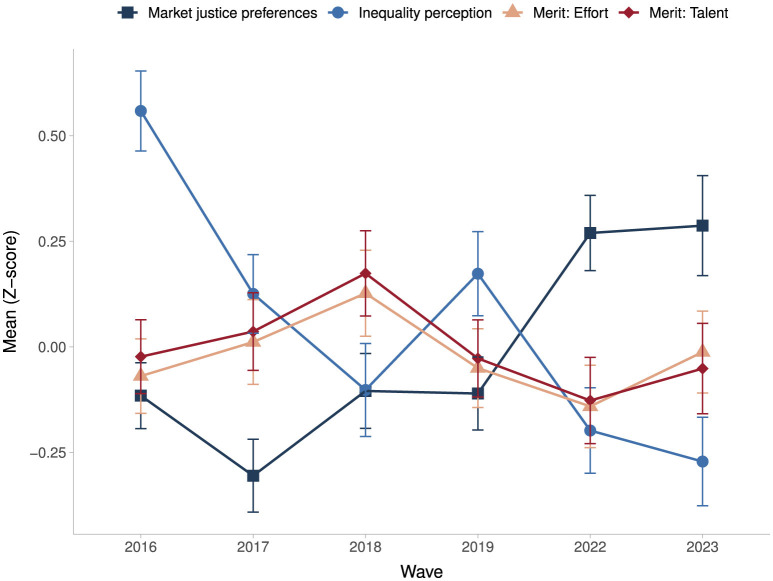
Change in the standarized mean of market justice preferences, economic inequality perception, and meritocracy (2016–2023). Source: own elaboration with pooled data from ELSOC 2016–2023 (*N* obs = 8,643; *N* groups = 1,687).

Regarding the main dependent and independent variables of this study, Figure 2 depicts their standardized average values across survey waves. The results show a notable upward trend in market justice preferences, particularly in the most recent waves. Perceived economic inequality exhibited the highest standardized mean in 2016; however, this variable shows a general downward trajectory over time, with a temporary increase in 2019, possibly reflecting the effects of the social uprising that year. Interestingly, while perceptions of inequality declined in the latest waves (2022–2023), market justice preferences continued to rise. Meritocratic perceptions, in contrast, remain relatively stable overall. Nevertheless, perceptions that individuals are rewarded based on talent tend to be slightly higher than those based on effort. Both meritocracy-related measures follow a similar temporal pattern: an increase from 2016 to 2018, a decline between 2019 and 2022—potentially associated with the social unrest and the consequences of the COVID-19 pandemic—and a subsequent rise in 2023, returning to levels comparable to those observed in 2016.

### 5.2 Multilevel models

Table 3 presents the results of the multilevel models estimated for market justice preferences, examining both individuals (within) and group-level (between) effects. The intraclass correlation (Hox et al., 2017) from the empty model (see Supplementary material), which decomposes the variance of market justice preferences, is 0.31, indicating that approximately 31% of the variation is attributable to differences between individuals. Complementary, 69% of the variation corresponds to within-individual differences over time.

**Table 3:** Longitudinal multilevel models for market justice preferences.

	**Model 1**	**Model 2**	**Model 3**	**Model 4**	**Model 5**	**Model 6**	**Model 7**
Intercept	1.938^***^	1.948^***^	1.965^***^	1.967^***^	1.974^***^	1.186^***^	1.250^***^
	(0.023)	(0.037)	(0.037)	(0.037)	(0.087)	(0.124)	(0.144)
Wave (Ref.= 2016)						
Wave 2017	−0.183^***^						
	(0.025)						
Wave 2018	−0.009						
	(0.025)						
Wave 2019	−0.009						
	(0.025)						
Wave 2022	0.300^***^						
	(0.025)						
Wave 2023	0.320^***^						
	(0.025)						
Wave		−0.088^***^	−0.095^***^	−0.096^***^	−0.096^***^	−0.096^***^	−0.096^***^
		(0.020)	(0.020)	(0.020)	(0.020)	(0.020)	(0.020)
Wave^2^		0.024^***^	0.024^***^	0.025^***^	0.025^***^	0.025^***^	0.025^***^
		(0.003)	(0.003)	(0.003)	(0.003)	(0.003)	(0.003)
Perception inequality (WE)			−0.027^**^	−0.025^**^	−0.025^**^	−0.025^**^	−0.025^**^
			(0.009)	(0.009)	(0.009)	(0.009)	(0.009)
Merit: effort (WE)				0.070^***^	0.070^***^	0.070^***^	0.070^***^
				(0.011)	(0.011)	(0.011)	(0.011)
Merit: talent (WE)				−0.027^*^	−0.027^*^	−0.027^*^	−0.027^*^
				(0.011)	(0.011)	(0.011)	(0.011)
Perception inequality (BE)					−0.002	0.043	0.008
					(0.023)	(0.023)	(0.024)
Merit: effort (BE)						0.206^***^	0.191^***^
						(0.041)	(0.040)
Merit: talent (BE)						0.036	0.021
						(0.040)	(0.040)
Controls	No	No	No	No	No	No	Yes
BIC	32, 146.711	31, 406.958	31, 414.699	31, 404.308	31, 419.062	31, 366.239	31, 473.850
Numb. obs.	8, 643	8, 643	8, 643	8, 643	8, 643	8, 643	8, 643
Num. groups: individuals	1, 687	1, 687	1, 687	1, 687	1, 687	1, 687	1, 687
Var: individuals (Intercept)	0.205	0.370	0.366	0.363	0.364	0.336	0.326
Var: residual	0.416	0.345	0.345	0.343	0.343	0.343	0.343
Var: individuals, wave		0.022	0.021	0.021	0.021	0.021	0.021
Cov: individuals (Intercept), wave		−0.061	−0.060	−0.059	−0.059	−0.058	−0.059

Cells contain regression coefficients with standard errors in parentheses. ^***^p < 0.001; ^**^p < 0.01; ^*^p < 0.05.

According to Model 1, which includes the survey waves to capture intertemporal variations in the dependent variable, there is a decrease in 2017 (β = -0.183, *p* < 0.001) relative to 2016, and similarly in 2018 (β = -0.009, *p* > 0.05) and 2019 (β = -0.009, *p* > 0.05), although the latter effects are not statistically significant. In contrast, in the more recent waves of 2022 and 2023, there is a statistically significant increase in market justice preferences (β = 0.300, *p* < 0.001; β = 0.320, *p* < 0.001), suggesting a non-linear effect. To model this trajectory over time, Model 2 incorporates time (survey waves) as a continuous variable, along with its quadratic term, representing the non-linear association initially observed in Model 1. While the linear term (survey wave) shows a negative association, reflecting an overall decline in market preferences over time, the positive quadratic term indicates a reversal of this pattern in the final measurement points.

Models 3 and 4 incorporate the within-group effects (WE) of the primary independent variables, capturing how individual changes in these variables over time shape the dependent variable. The results in Model 3 suggest that the within effect of perceived economic inequality is negative and statistically significant (*p* < 0.001). Specifically, each one-point increase in an individual's perception of economic inequality between waves is associated with a 0.027 point decrease in market justice preferences. Model 4 shows that meritocratic perceptions operate in distinct directions. An upward shift in the perception that effort is rewarded exerts a positive within effect (β = 0.070, *p* < 0.001), while a parallel increase in the perception that intelligence and ability are rewarded is likewise associated with lower market-justice preferences (β = -0.027, *p* < 0.05). Taken together, these results suggest that people who increasingly perceive meritocracy based on effort tend to have stronger preferences for market justice, and that the opposite is true for those who increasingly perceive meritocracy based on talent.

When examining the between-group effects (BE) in Model 5 and 6, which capture differences between individuals in the average of the main variables, a similar pattern emerges. Individuals who perceive higher levels of economic inequality tend to prefer less market justice (β = -0.002, *p* > 0.05). However, this effect is no longer statistically significant. In Model 6, the meritocratic perception that effort is rewarded is positively associated with market justice preferences (β = 0.206, *p* < 0.001), whereas the perception that talent is rewarded shows a positive but non-significant coefficient (β = 0.036, *p* > 0.05).

Model 7 adds the control variables. The within- and between-effects of the principal predictors retain both their direction and statistical significance, confirming the robustness of the associations (see Supplementary material for effects of control variables).

Table 4 examines whether perceived economic inequality moderates the effect of meritocratic perceptions on market justice preferences. Contrary to our expectations, the interaction terms in the within-person specification of Model 9 indicate that the negative effect of talent-based meritocratic perceptions becomes stronger as perceived inequality increases (β = –0.032, *p* < 0.05). In contrast, in the between-person specification (Model 11), the interaction term is positive (β = 0.099, *p* < 0.01), suggesting that perceptions of economic inequality significantly shape the influence of meritocratic perceptions on support for market-based allocation of social services. Specifically, these positive effects indicate that, as perceptions of inequality increase, the association between talent-based meritocratic perceptions and market justice preferences shifts in a positive direction.

**Table 4:** Interactions for meritocracy, perceived economic inequality and market justice preferences.

	**Model 8**	**Model 9**	**Model 10**	**Model 11**
Intercept	1.328^***^	1.346^***^	1.956^***^	2.281^***^
	(0.143)	(0.143)	(0.350)	(0.369)
Perception inequality (WE)	−0.037^*^	−0.036^*^	−0.040^***^	−0.040^***^
	(0.015)	(0.015)	(0.009)	(0.009)
Merit: effort (WE)	0.075^***^	0.085^***^	0.081^***^	0.081^***^
	(0.018)	(0.012)	(0.011)	(0.011)
Merit: talent (WE)	−0.021	−0.017	−0.026^*^	−0.026^*^
	(0.011)	(0.016)	(0.011)	(0.011)
Perception inequality (BE)	−0.003	−0.003	−0.176	−0.268^**^
	(0.023)	(0.023)	(0.096)	(0.101)
Merit: effort (BE)	0.180^***^	0.183^***^	−0.045	0.191^***^
	(0.040)	(0.040)	(0.127)	(0.041)
Merit: talent (BE)	0.022	0.018	0.017	−0.320^*^
	(0.039)	(0.039)	(0.041)	(0.129)
Merit: effort (WE) × perception inequality (WE)	−0.014			
	(0.013)			
Merit: talent (WE) × perception inequality (WE)		−0.032^*^		
		(0.013)		
Merit: effort (BE) × perception inequality (BE)			0.070	
			(0.036)	
Merit: talent (BE) × perception inequality (BE)				0.099^**^
				(0.036)
Controls	Yes	Yes	Yes	Yes
BIC	31, 201.714	31, 254.484	32, 281.698	32, 277.809
Numb. obs.	8, 643	8, 643	8, 643	8, 643
Num. groups: individuals	1, 687	1, 687	1, 687	1, 687
Var: individuals (intercept)	0.178	0.178	0.170	0.169
Var: individuals, merit effort cwc	0.127			
Var: individuals, perception inequality cwc	0.090	0.088		
Cov: individuals (intercept), merit effort cwc	−0.009			
Cov: individuals (intercept), perception inequality cwc	−0.015	−0.013		
Cov: individuals, merit effort cwc, perception inequality cwc	0.003			
Var: residuals	0.296	0.303	0.417	0.417
Var: individuals, merit talent cwc		0.097		
Cov: individuals (intercept), merit talent cwc		−0.011		
Cov: individuals, merit talent cwc, perception inequality cwc		−0.004		

Cells contain regression coefficients with standard errors in parentheses. ^***^p < 0.001; ^**^p < 0.01; ^*^p < 0.05. CWC, centered within group.

**Figure 3:**
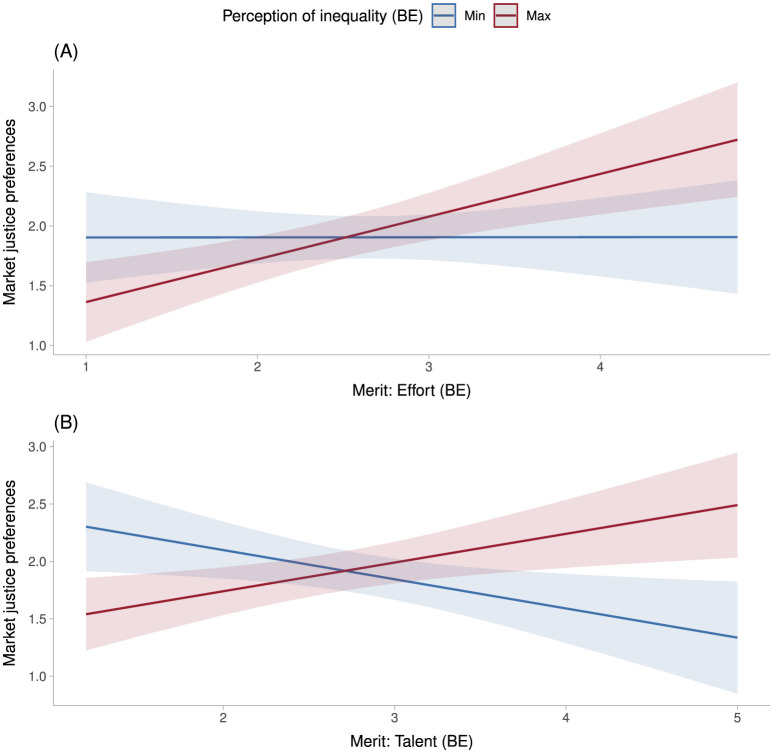
Predicted values of market justice preferences by perceptions of meritocraticy and economic inequality. **(A)** People are rewarded by their efforts. **(B)** People are rewarded for their intelligence. Source: own elaboration with data from ELSOC 2016–2023 (n = 8,460). Based in Model 10 and Model 11 in Table 4.

The effects of effort-based meritocratic perceptions are not statistically significant in either the within- or between-person specifications (Models 8 and 10). However, the direction of the coefficients is negative in the former and positive in the latter. As shown in Figure 3A, the between-person effect of effort-based meritocratic perceptions on market justice preferences strengthens as perceived inequality increases across individuals, whereas this effect weakens among those who perceive lower levels of inequality. Figure 3B reveals a similar pattern for the between-person effect of talent-based meritocratic perceptions, although the effect is substantially weaker at low levels of perceived inequality.

Substantively, these findings suggest that the legitimizing function of meritocratic perceptions is amplified in contexts perceived as highly unequal. In such contexts, meritocratic narratives may reinforce individualistic understandings of distributive justice and intensify support for market-based access to essential social services.

Regarding the temporal dynamics of the key predictors, the analysis shows that time has no statistically significant effects on the intrapersonal or interpersonal effects of perceived inequality and meritocratic perceptions. Further details about this analysis can be found in Supplementary material.

## 6 Discussion

The first set of hypotheses proposed that perceptions of economic inequality are strongly associated with preferences for market justice. In the between-individual specification, the effect of perceived inequality is negative but not statistically significant. Therefore, we cannot conclude that individuals who, on average, perceive higher levels of inequality are systematically less supportive of market justice. As a result, hypothesis *H*1_*a*_ is not supported by the data. However, in the within-individual specification, the findings reveal a significant negative association: individuals who, at a given point in time, perceive higher income disparities are less supportive of the idea that access to core social services should depend on individual income. This result aligns with theories suggesting that increased awareness of inequality fosters a more critical stance toward market-based distributive arrangements (Castillo, 2012; García-Sánchez et al., 2019; Mijs, 2021). Such a pattern reflects a moral economy logic in which perceptions of systemic unfairness undermine the legitimacy of existing distributions and strengthen demands for greater equity. Specifically, the negative within-individual effect over time (*H*1_*b*_) suggests that as inequality becomes more salient for some individuals, their support for market justice declines—possibly due to growing distrust in market mechanisms or increasing disillusionment with the perceived fairness of the system.

Regarding the second set of hypotheses, the results confirmed that meritocratic perceptions—particularly those emphasizing individual effort—were associated with stronger support for market-based distribution systems. Individuals who believed that success is primarily achieved through personal effort were more likely to justify unequal access to core social services based on income, interpreting such disparities as outcomes of individual merit rather than systemic injustice (*H*2_*a*_). This finding aligns with previous research showing that meritocratic narratives serve as moral justifications that legitimize social stratification (Castillo, 2012; Hoyt et al., 2023). These perceptions operate symbolically to reinforce structural inequalities by reducing support for redistributive policies, framing inequality as both fair and deserved. Consistent with prior work by Castillo et al. (2024) on Chilean students, the results suggest that such meritocratic perceptions uphold existing hierarchies by promoting the acceptance of inequality as a reflection of individual virtue rather than structural failure. This mechanism is particularly salient in neoliberal contexts like Chile, where market logics heavily shape social attitudes (Canales Cerón et al., 2021).

Interestingly, intra-individual changes in meritocratic perceptions over time (*H*2_*b*_) reveal mixed effects. While increases in the belief that rewards are based on individual effort are associated with stronger preferences for market justice, increases in the belief that rewards derive from talent are linked to weaker support for such principles. One possible explanation is that effort is generally viewed as a controllable and malleable trait, whereas talent tends to be perceived as innate and less subject to personal control, rendering talent-based inequality less legitimate. Furthermore, increased exposure to real-world scenarios in which outcomes are clearly shaped by inherent traits rather than hard work may lead individuals to question the fairness and legitimacy of market-based reward systems.

The third set of hypotheses addressed the moderating role of perceptions of inequality in the relationship between meritocratic perceptions and preferences for market justice. The analysis revealed that the positive association between meritocratic perceptions and support for market-based distribution systems tends to be stronger when perceived economic inequality is high, contradicting our initial expectations (*H*3_*a*_ and *H*3_*b*_). Although this moderating effect is not statistically significant for effort-related meritocratic perceptions, it is significant for talent-related perceptions at both the within- and between-individual levels. However, this interpretation requires caution: the main effect of perceived inequality on support for market justice is negative, meaning that as perceived inequality becomes less negative (i.e., closer to zero), the positive relationship between talent-based meritocratic perceptions and market justice preferences becomes stronger. This suggests a more nuanced dynamic: meritocratic perceptions, particularly those emphasizing talent, may serve as a stronger justificatory mechanism for market-based inequalities among individuals who are less inclined to perceive inequality as a problem. In other words, among those who do not strongly perceive systemic disparities, meritocratic narratives may play a more influential role in legitimizing unequal outcomes.

Finally, the fourth exploratory hypothesis examined the potential impact of social events—particularly those that erupted in 2019—on the relationship between meritocratic perceptions, perceptions of inequality, and preferences for market justice. The main effect of time indicates that, on average, support for market justice increased after 2019 protests. However, the analysis did not detect any significant interactions between distributive beliefs and support for market justice, suggesting that events in this period (as the mobilizations, the pandemic and/or the constitucional processes) did not substantially modify the underlying associations between these variables. As a result, hypotheses *H*4_*a*_ and *H*4_*b*_ are not supported by the analysis. This may point to a certain stability or even resilience in the normative frameworks that guide individuals' evaluations of distributive justice, despite the occurrence of major collective political events. Alternatively, while the protests may have eroded trust in institutional arrangements or governance, they may not have fundamentally altered individuals' beliefs about how rewards and resources should be allocated in society.

## 7 Conclusions

This study examined the complex interplay between perceptions of economic inequality, meritocratic perceptions, and preferences toward market justice in Chile from 2016 to 2023, drawing on longitudinal data from the ELSOC survey. By exploring how subjective assessments and social contexts influence support for redistribution and market-based resource allocation, the research offers different elements that contribute to the understanding of the normative foundations underpinning social justice attitudes in a highly unequal and commodified environment.

The findings support that higher perceptions of economic inequality are associated with less support for market justice preferences, this is, the belief that it is fair that those with higher income have better social services such as education, pensions and health. At the same time, meritocratic perceptions—particularly those emphasizing individual effort—are strongly associated with support for market-based distributions, suggesting that meritocracy serve as a moral justification for structural inequalities. However, changes in meritocratic perceptions over time reveal a more nuanced picture: while increased emphasis on effort reinforces support for market justice, increased emphasis on talent tends to reduce it. This distinction likely reflects broader beliefs about the controllability and fairness of different meritocratic traits. Moreover, the interaction between inequality perceptions and meritocratic perceptions indicates that the legitimizing power of meritocracy becomes stronger as perceptions of inequality become less negative—that is, when individuals are less aware or concerned about economic disparities. Such finding highlights a potential feedback mechanism by which lower sensitivity to inequality may enable stronger endorsement of merit-based explanations for unequal outcomes.

This research advances the extant literature by integrating subjective perceptions with social and political contexts to explain attitudes toward economic inequality and distributional justice. While previous studies primarily focused on objective measures or individual characteristics (Busemeyer, 2014; Immergut and Schneider, 2020; Lindh, 2015), this work emphasizes the dynamic and interactional nature of perceptions and beliefs over time. Furthermore, it highlights the importance of socio-political upheavals in reshaping normative attitudes, underscoring the role of collective action in challenging entrenched narratives of meritocracy and fairness. The longitudinal approach provides a deeper temporal perspective on how societal events could influence individual perceptions and preferences.

Regarding avenues for future research, experimental evidence could help expand the understanding of the association between perceptual variables and market justice preferences. International comparative studies are also necessary in order to assess the role of national contexts and the universality or specificity of these dynamics. Additionally, investigating the role of media, political communication, and education in shaping perceptions of inequality and meritocracy would deepen understanding of the normative foundations of social justice attitudes and their variation over time. Finally, examining how these perceptions influence behavioral outcomes, such as political participation or support for social movements, would provide valuable insights into the pathways from beliefs to collective action and policy change.

## Data Availability

Publicly available datasets were analyzed in this study. This data can be found at: https://dataverse.harvard.edu/dataverse/elsoc.
