# Supplementary material for: Perceptions of inequality and meritocracy: their interplay in shaping preferences for market justice in Chile (2016–2023)
Source: Front Sociol. 2025 Sep 18;10:1634219. doi: 10.3389/fsoc.2025.1634219 (PMC12488697; doi:10.3389/fsoc.2025.1634219)
Supplement: Supplementary file 1 [file Data_Sheet_1.pdf]

Supplementary material

Table 1: Descriptive statistics for control variables for the first wave (2016)

Label	Stats / Values	Freqs (% of Valid)	Valid
Educational level	1. Less than Universitary	1242 (82.7%)	1501
	2. Universitary	259 (17.3%)	(100.0%)
Household income quintile (per capita)	1. Q1	323 (21.5%)	1501
	2. Q2	291 (19.4%)	(100.0%)
	3. Q3	290 (19.3%)	
	4. Q4	285 (19.0%)	
	5. Q5	254 (16.9%)	
	6. QNA	58 (3.9%)	
Sex	1. Male	535 (35.6%)	1501
	2. Female	966 (64.4%)	(100.0%)
Age	1. 18-29	219 (14.6%)	1501
	2. 30-49	603 (40.2%)	(100.0%)
	3. 50-64	477 (31.8%)	
	4. 65 or more	202 (13.5%)	
Subjective social status	Mean (sd) : 4.3 (1.5)	11 distinct values	1501
	min < med < max:		(100.0%)
	0 < 4 < 10		
	IQR (CV) : 2 (0.3)		
Political identification	1. Left	325 (21.7%)	1501
	2. Center	320 (21.3%)	(100.0%)
	3. Right	201 (13.4%)	
	4. Does not identify	655 (43.6%)	

Figure 1: Correlation matrix of the main variables for the first wave (2016)

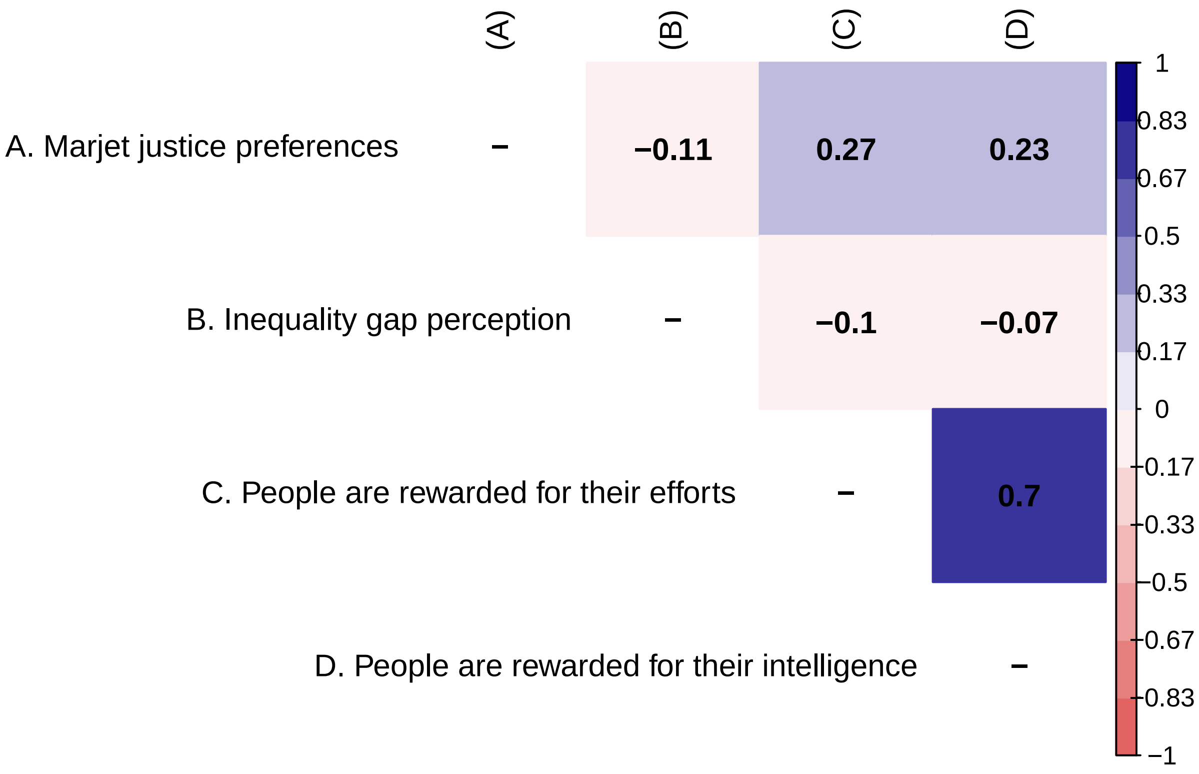

Source: own elaboration with data from ELSOC 2016 (n = 1687)

Table 2: Complete longitudinal multilevel models for market justice preferences

	Model 0	Model 1	Model 2	Model 3	Model 4	Model 5	Model 6	Model 7
Intercept	2.010^{***}	1.938^{***}	1.948^{***}	1.965^{***}	1.967^{***}	1.974^{***}	1.186^{***}	1.250^{***}
	(0.017)	(0.023)	(0.037)	(0.037)	(0.037)	(0.087)	(0.124)	(0.144)
Wave (Ref.= 2016)								
Wave 2017		-0.183^{***}						
		(0.025)						
Wave 2018		-0.009						
		(0.025)						
Wave 2019		-0.009						
		(0.025)						
Wave 2022		0.300^{***}						
		(0.025)						
Wave 2023		0.320^{***}						
		(0.025)						
Wave			-0.088^{***}	-0.095^{***}	-0.096^{***}	-0.096^{***}	-0.096^{***}	-0.096^{***}
			(0.020)	(0.020)	(0.020)	(0.020)	(0.020)	(0.020)
Wave^2			0.024^{***}	0.024^{***}	0.025^{***}	0.025^{***}	0.025^{***}	0.025^{***}
			(0.003)	(0.003)	(0.003)	(0.003)	(0.003)	(0.003)
Perception inequality (WE)				-0.027^{**}	-0.025^{**}	-0.025^{**}	-0.025^{**}	-0.025^{**}
				(0.009)	(0.009)	(0.009)	(0.009)	(0.009)
Merit: Effort (WE)					0.070^{***}	0.070^{***}	0.070^{***}	0.070^{***}
					(0.011)	(0.011)	(0.011)	(0.011)
Merit: Talent (WE)					-0.027^{*}	-0.027^{*}	-0.027^{*}	-0.027^{*}
					(0.011)	(0.011)	(0.011)	(0.011)
Perception inequality (BE)						-0.002	0.043	0.008
						(0.023)	(0.023)	(0.024)
Merit: Effort (BE)							0.206^{***}	0.191^{***}
							(0.041)	(0.040)
Merit: Talent (BE)							0.036	0.021
							(0.040)	(0.040)
University education (Ref.= Less than University)								0.003
								(0.043)
Income quintile (Ref.= Quintile 1)								
Quintile Q2								-0.004
								(0.051)
Quintile Q3								0.078
								(0.050)
Quintile Q4								0.115^{*}
								(0.051)
Quintile Q5								0.184^{***}
								(0.054)
Quintile no information								0.217^{**}
								(0.077)
Subjective social status								-0.002
								(0.011)
Political identification (Ref.= Left)								
Center								0.111^{*}
								(0.044)
Right								0.334^{***}
								(0.052)
Does not identify								0.074
								(0.041)
Female (Ref.= Male)								-0.095^{**}
								(0.032)
Age (Ref.= 18-29)								
Age 30-49								-0.014
								(0.044)
Age 50-64								0.031
								(0.047)
Age 65 or more								0.068
								(0.057)
BIC	32681.306	32146.711	31406.958	31414.699	31404.308	31419.062	31366.239	31473.850
Numb. obs.	8643	8643	8643	8643	8643	8643	8643	8643
Num. groups: individuals	1687	1687	1687	1687	1687	1687	1687	1687
Var: individuals (Intercept)	0.203	0.205	0.370	0.366	0.363	0.364	0.336	0.326
Var: Residual	0.449	0.416	0.345	0.345	0.343	0.343	0.343	0.343
Var: individuals, wave			0.022	0.021	0.021	0.021	0.021	0.021
Cov: individuals (Intercept), wave			-0.061	-0.060	-0.059	-0.059	-0.058	-0.059

Note: Cells contain regression coefficients with standard errors in parentheses. $^{***}p < 0.001$; $^{**}p < 0.01$; $^{*}p < 0.05$.

Source: own elaboration with pooled data from ELSOC 2016-2023 (N obs = 8643; N groups = 1687)

Table 3: Growth curves for meritocracy, perceived economic inequality and market justice preferences

	Model 12	Model 13	Model 14	Model 15	Model 16	Model 17
Intercept	1.263***	1.264***	1.246***	1.128***	1.099***	1.090***
	(0.143)	(0.141)	(0.142)	(0.176)	(0.169)	(0.170)
Wave x Perception inequality (WE)	0.003					
	(0.006)					
Wave x Merit: effort (WE)		0.012				
		(0.006)				
Wave x Merit: talent (WE)			0.007			
			(0.006)			
Wave x Perception inequality (BE)				−0.011		
				(0.009)		
Wave x Merit: effort (BE)					−0.020	
					(0.011)	
Wave x Merit: talent (BE)						−0.016
						(0.010)
Controls	Yes	Yes	Yes	Yes	Yes	Yes
BIC	31010.853	30888.601	30785.269	31515.978	31493.536	31501.405
Numb. obs.	8643	8643	8643	8643	8643	8643
Num. groups: individuals	1687	1687	1687	1687	1687	1687
Var: individuals (Intercept)	0.388	0.334	0.379	0.274	0.535	0.372
Var: individuals, perception inequality cwc	0.100					
Var: individuals, wave	0.025	0.023	0.024	0.021	0.021	0.021
Cov: individuals (Intercept), perception inequality cwc	−0.081					
Cov: individuals (Intercept), wave	−0.074	−0.062	−0.072	−0.054	−0.056	−0.049
Cov: individuals, perception inequality cwc, wave	0.021					
Var: Residuals	0.291	0.280	0.275	0.343	0.342	0.343
Var: individuals, merit effort cwc		0.122				
Cov: individuals (Intercept), merit effort cwc		−0.009				
Cov: individuals, merit effort cwc, wave		0.000				
Var: individuals, merit talent cwc			0.112			
Cov: individuals (Intercept), merit talent cwc			0.003			
Cov: individuals, merit talent cwc, wave			−0.003			
Var: individuals, perception inequality mean				0.000		
Cov: individuals (Intercept), perception inequality mean				0.007		
Cov: individuals, perception inequality mean, wave				−0.001		
Var: individuals, merit effort mean					0.051	
Cov: individuals (Intercept), merit effort mean					−0.110	
Cov: individuals, merit effort mean, wave					−0.001	
Var: individuals, merit talent mean						0.031
Cov: individuals (Intercept), merit talent mean						−0.053
Cov: individuals, merit talent mean, wave						−0.003

Note: Cells contain regression coefficients with standard errors in parentheses. $^{***}p < 0.001$; $^{**}p < 0.01$; $^{*}p < 0.05$. CWC = centered within group.

Source: own elaboration with pooled data from ELSOC 2016-2023 (N obs = 8643; N groups = 1687)

Table 4: Interactions for meritocracy, perceived economic inequality and market justice preferences

	Model 18	Model 19	Model 20	Model 21	Model 22	Model 23
Intercept	1.104***	1.124***	1.150***	0.791**	0.785***	0.989***
	(0.146)	(0.144)	(0.144)	(0.244)	(0.224)	(0.231)
Wave 2017 x Perception inequality (WE)	−0.044					
	(0.036)					
Wave 2018 x Perception inequality (WE)	0.083*					
	(0.034)					
Wave 2019 x Perception inequality (WE)	0.035					
	(0.034)					
Wave 2022 x Perception inequality (WE)	0.090*					
	(0.036)					
Wave 2023 x Perception inequality (WE)	0.002					
	(0.036)					
Wave 2017 x Merit: effort (WE)		−0.087*				
		(0.036)				
Wave 2018 x Merit: effort (WE)		−0.044				
		(0.036)				
Wave 2019 x Merit: effort (WE)		0.046				
		(0.037)				
Wave 2022 x Merit: effort (WE)		−0.043				
		(0.038)				
Wave 2023 x Merit: effort (WE)		0.047				
		(0.038)				
Wave 2017 x Merit: talent (WE)			−0.157***			
			(0.035)			
Wave 2018 x Merit: talent (WE)			−0.121***			
			(0.036)			
Wave 2019 x Merit: talent (WE)			−0.031			
			(0.035)			
Wave 2022 x Merit: talent (WE)			−0.133***			
			(0.036)			
Wave 2023 x Merit: talent (WE)			0.003			
			(0.036)			
Wave 2017 x Perception inequality (BE)				−0.046		
				(0.034)		
Wave 2018 x Perception inequality (BE)				−0.016		
				(0.035)		
Wave 2019 x Perception inequality (BE)				−0.007		
				(0.040)		
Wave 2022 x Perception inequality (BE)				−0.078		
				(0.045)		
Wave 2023 x Perception inequality (BE)				−0.057		
				(0.049)		
Wave 2017 x Merit: effort (BE)					−0.082*	
					(0.039)	
Wave 2018 x Merit: effort (BE)					−0.040	
					(0.041)	
Wave 2019 x Merit: effort (BE)					−0.055	
					(0.045)	
Wave 2022 x Merit: effort (BE)					−0.119*	
					(0.050)	
Wave 2023 x Merit: effort (BE)					−0.119*	
					(0.056)	
Wave 2017 x Merit: talent (BE)						0.024
						(0.038)
Wave 2018 x Merit: talent (BE)						−0.006
						(0.040)
Wave 2019 x Merit: talent (BE)						−0.064
						(0.044)
Wave 2022 x Merit: talent (BE)						−0.048
						(0.050)
Wave 2023 x Merit: talent (BE)						−0.058
						(0.055)
Controls	Yes	Yes	Yes	Yes	Yes	Yes
BIC	30936.087	30829.408	30711.660	31474.882	31459.508	31462.070
Num. obs.	8643	8643	8643	8643	8643	8643
Num. groups: idencuesta	1687	1687	1687	1687	1687	1687
Var: idencuesta (Intercept)	0.388	0.343	0.388	0.277	0.122	0.405
Var: idencuesta perc_inequality_cwc	0.101					
Var: idencuesta ola_num	0.025	0.023	0.025	0.021	0.021	0.021
Cov: idencuesta (Intercept) perc_inequality_cwc	−0.079					
Cov: idencuesta (Intercept) ola_num	−0.073	−0.063	−0.074	−0.054	−0.036	−0.050
Cov: idencuesta perc_inequality_cwc ola_num	0.020					
Var: Residual	0.284	0.273	0.268	0.336	0.336	0.336
Var: idencuesta merit_effort_cwc		0.123				
Cov: idencuesta (Intercept) merit_effort_cwc		−0.008				
Cov: idencuesta merit_effort_cwc ola_num		0.001				
Var: idencuesta merit_talent_cwc			0.111			
Cov: idencuesta (Intercept) merit_talent_cwc			0.000			
Cov: idencuesta merit_talent_cwc ola_num			−0.002			
Var: idencuesta perc_inequality_mean				0.000		
Cov: idencuesta (Intercept) perc_inequality_mean				0.007		
Cov: idencuesta perc_inequality_mean ola_num				−0.001		
Var: idencuesta merit_effort_mean					0.008	
Cov: idencuesta (Intercept) merit_effort_mean					0.030	
Cov: idencuesta merit_effort_mean ola_num					−0.009	
Var: idencuesta merit_talent_mean						0.034
Cov: idencuesta (Intercept) merit_talent_mean						−0.063
Cov: idencuesta merit_talent_mean ola_num						−0.003

Note: Cells contain regression coefficients with standard errors in parentheses. $^{***}p < 0.001$; $^{**}p < 0.01$; $^{*}p < 0.05$. CWC = centered within group.

Source: own elaboration with pooled data from ELSOC 2016-2023 (N obs = 8643; N groups = 1687)
